# PARE: A tool for comparing protein abundance and mRNA expression data

**DOI:** 10.1186/1471-2105-8-309

**Published:** 2007-08-24

**Authors:** Eric Z Yu, Anne E Counterman Burba, Mark Gerstein

**Affiliations:** 1 Program in Computational Biology and Bioinformatics, Yale University, New Haven, CT 06520, USA; 2 Department of Molecular Biophysics and Biochemistry, Yale University, New Haven, CT 06520, USA; 3 Department of Computer Science, Yale University, New Haven, CT 06520, USA

## Abstract

**Background:**

Techniques for measuring protein abundance are rapidly advancing and we are now in a situation where we anticipate many protein abundance data sets will be available in the near future. Since proteins are translated from mRNAs, their expression is expected to be related to their abundance, to some degree.

**Results:**

We have developed a web tool, called PARE (Protein Abundance and mRNA Expression; ), to correlate these two quantities. In addition to globally comparing the quantities of protein and mRNA, PARE allows users to select subsets of proteins for focused study (based on functional categories and complexes). Furthermore, it highlights correlation outliers, which are potentially worth further examination.

**Conclusion:**

We anticipate PARE will facilitate comparative studies on mRNA and protein abundance by the proteomics community.

## Background

Quantifying mRNA expression is currently one of the most exciting and active areas in genomics, and as a result there are copious amounts of data available. However, mRNA does not directly arbitrate biological function; that role is filled by proteins, which are translated from mRNA and connect mRNA to biological processes by acting on the latter as a regulator. In contrast to the abundance of mRNA expression data, considerably fewer quantitative protein expression datasets are available due to the comparatively recent advent of methods for measuring protein abundance on a large scale (such as the use of ICAT and iTRAQ with mass spectrometry) and the difficulty of these experiments relative to chip-based mRNA studies. Also, for many studies, protein abundance data has not been published and is not available via public databases. Because protein levels are linked to mRNA expression by the process of translation, we expect a relationship between these abundances. Moreover, if a high correlation is found, we can use mRNA expression data to directly model protein expression. Conversely, a low correlation indicates that the abundance of a particular protein is somewhat independent of mRNA expression. Similar to the first-order kinetics theory of chemical reactions, the theory governing the relation between mRNA and protein considers the protein synthesis rate to be proportional to the corresponding mRNA concentration and the protein degradation rate to be proportional to protein concentration [1]. This relationship can be expressed in the equation: *d [P]*_*i*_/*dt *= *k*_*s*, *i*_*[mRNA]*_*i*_-*k*_*d*, *i*_*[P]*_*i*_

where [P]_i _is the concentration of protein i, [mRNA]_i _is the corresponding mRNA concentration, k_s, i _is the protein synthesis rate constant, and k_d, i _is the overall protein degradation and dilution rate constant [2-4]. Ideally, a time series of protein abundance and corresponding mRNA expression data could be used to verify the relationship in the above equation, but such experiments are hard to implement. However, at steady state, the change of protein abundance over time (the left side of the above equation) can be assumed to be zero, giving *[P]*_*i *_= (*k*_*s*, *i*_/*k*_*d*, *i*_*) [mRNA]*_*i*_.

This equation suggests that a linear correlation between protein abundance and mRNA expression level is expected at steady state. To date, there have been a small number of studies to correlate experimental mRNA expression levels and protein abundance, mostly in human cancer and yeast cells. For the most part, only limited correlations are reported [5-10]. These results suggest that complicated post-transcriptional and/or post-translational mechanisms may be involved in determining final protein abundance.

Note that in the equations, the rate constants k_s, i _and k_d, i _vary by protein species. Thus, in addition to a global correlation, a more reasonable analysis will focus on a given protein species and study a time series of its abundance with the corresponding mRNA expression, or focus on members of a protein complex, which are likely to have similar rate constants, at a steady state. Greenbaum et al. found a significantly higher correlation for proteins with high ribosomal occupancy (i.e., much of the expressed mRNA in the cell is associated with ribosomes and therefore being translated) and high variability (associated with highly controlled mRNA regulation), respectively [9]. Furthermore, there might be a significant amount of error and noise, intrinsic and extrinsic, in both protein and mRNA experiments [11-13]. This problem is expected to be alleviated by advances in experimental techniques and improvements in data quality.

## Implementation

We have developed a tool to conduct the aforementioned studies on a large scale in order to advance our understanding of the relationship between protein and mRNA expression. The package, named PARE, is implemented in Perl/CGI on our website and available to the research community [14]. The server operates on GNU/Linux 2.6, and runs Apache 2.0.

The implementation of the web tool can be divided into three parts: (a) selection of mRNA and protein abundance data; (b) correlating mRNA and protein data for selected subsets; and (c) identifying outliers from the trend. Each of these parts is described in more detail below.

### a. Selection of mRNA and protein abundance data

PARE requires as input quantitative mRNA and protein abundance data. On our website, users can choose to upload these datasets, select from pre-collected, currently available datasets (Figure 1 of Supplementary Data), or obtain datasets from external databases. Upon entering the web tool, the user is directed to either select an organism to access the corresponding pre-collected datasets or to upload datasets. We note that there are many mRNA microarray datasets available at external databases, including Symatlas [15], NCBI Gene Expression Omnibus (GEO) [16,17], and Yale Protein Expression Database (YPED). We provide links to these sites from PARE, and are in the process of developing a direct interface for accessing and analyzing YPED datasets.

**Figure 1:**
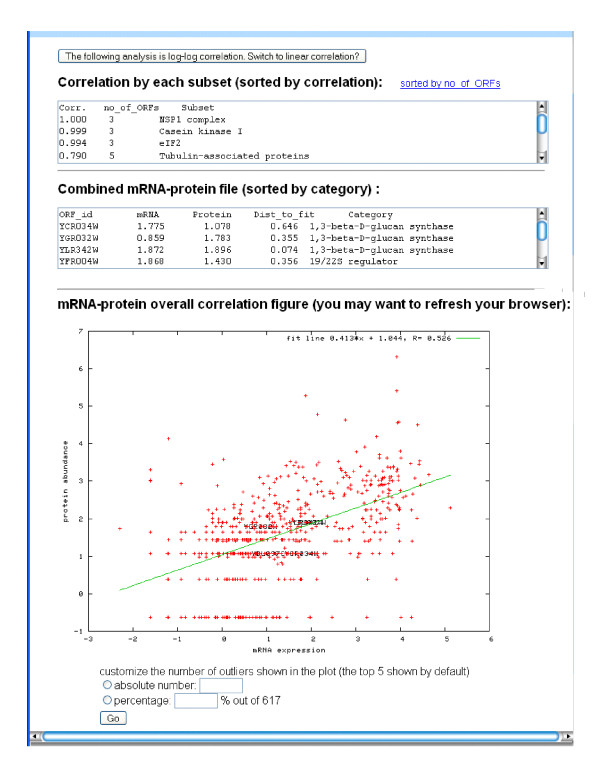
Example output from PARE for a categorization analysis.

The available pre-collected datasets include large-scale yeast data [7,8,18-24], as well as a small amount of mammalian data. Relative quantitation datasets (i.e., expression or abundance *ratios*) are also included, and are denoted on the site by 'REL'. These datasets are typically correlated on a log-log scale. To date, we have included only published datasets as those that are pre-collected.

A complete list of citations for the pre-collected datasets is available online [14]. This table details the data type, organism, number of ORFs for which data are available, experimental method, whether the dataset is relative (ratios) or absolute quantitation, and PubMed-linked citations. We expect the collected data to increase considerably in the future.

### b. Correlating mRNA and protein data for selected subsets

An overall correlation between mRNA and protein is useful to give a sense of the big picture, and users can choose "*correlate everything*" to conduct this analysis. Users can also perform the correlation for a subset of proteins selected from MIPS [25] or Gene Ontology (GO) [26] categories, or upload a definition file to use a customized categorization. Once the user has selected subcategories for analysis, MySQL is used to retrieve the corresponding mRNA and protein data for correlation analysis. If a categorization analysis is chosen, the correlation from all the selected categories will be shown, sorted by correlation (default) or by category size (example output shown in Figure 1). GNUplot is used to generate the correlation plots. We also include the value for mutual information computed from the mRNA expression and protein abundance levels. This quantity will highlight relationships between mRNA and protein that are not linear. (The default number of bins used for the mutual information calculation is taken to be ten percent of the number of matched pairs of mRNA and protein levels).

### c. Identifying outliers from the trend

After the proteins of interest are specified by users, PARE outputs the mRNA-protein scatter plot where the correlation, fit line and its equation, and the top outliers will be highlighted, as well as the combined mRNA-protein abundance data sorted by the perpendicular distance of a data point to the fit line. An example of the output is shown in Figure 2 of the supplementary data [see Additional file 1]. A few options are available for user customization of the output. In some cases, such as when the data are highly scattered, a log-log correlation will make more sense than the default linear correlation, and users can instruct PARE to toggle to a log-log plot. Additionally, the user can customize the number of the outliers shown (the default number is 5) by absolute number or relative percentage.

## Results and discussion

PARE provides a rapid means of quickly assessing correlations in quantitative proteomics data for matched experimental mRNA and protein abundance datasets. It cannot be overemphasized that the quality of the correlations obtained is dependent upon appropriate selection of corresponding mRNA expression and protein abundance datasets. The user bears the responsibility of determining whether the experimental conditions for uploaded mRNA and protein datasets are sufficiently analogous to merit correlation. Another aspect for users to consider when reviewing datasets is any pre-processing steps that occurred between data collection and tabulation of quantitative expression or abundance values or ratios.

In biological systems, there are clearly many factors that may influence the correlation between mRNA expression and protein abundance. Protein synthesis and degradation rates, post-transcriptional mRNA regulation, and even experimental noise can affect the results. The correlation analysis provided by PARE allows us to identify targets for more detailed study to further the development of new models.

A key feature of our tool is the ability to perform the correlation analysis for selected subcategories (and groupings thereof) of data. The correlations for different GO categories can, in fact, be dramatically different (see Table 1, Supplementary Data). As discussed in the Introduction, this analysis will provide an insight into how closely-connected proteins are co-translated.

The correlation outliers are key targets for further experimental studies. Tracing the expression and translation processes of the outliers in detail should lead us to an understanding of why they do not correlate well (perhaps because of slow protein degradation for particular proteins) and the underlying biological processes involved.

## Conclusion

We have developed a web tool, PARE, to analyze protein abundance and mRNA expression data. The utility of the correlations provided by PARE will improve as the quality of available data sets increases and the methodologies for determining protein abundance are refined. For instance, current protein abundance data are deduced from the intensity of gel spots or the measurement of identified peptides from enzymatic digests, yet Ishihama et al. shows that emPAI (exponentially modified protein abundance index) may be a better parameter to use [27]. We anticipate PARE will facilitate comparative studies on mRNA and protein abundance by the proteomics community.

## Availability

Project name: PARE

**Project home page**: 

**Contact**: proteomics@bioinfo.mbb.yale.edu

**Operating systems**: Platform independent

**Programming language**: Perl/CGI

## Authors' contributions

EY and ACB designed and developed the PARE tool. MG conceived the project and supervised the development and implementation of the tool. EY, ACB, and MG drafted the manuscript. All authors read and approved the manuscript.

## Supplementary Material

Additional file 1 Document includes two figures: (1) a screenshot of the PARE web site; and (2) a screenshot of the interactive analysis results page. Click here for file
