# Supplementary material for: PARE: A tool for comparing protein abundance and mRNA expression data
Source: BMC Bioinformatics. 2007 Aug 24;8:309. doi: 10.1186/1471-2105-8-309 (PMC2000916; doi:10.1186/1471-2105-8-309)
Supplement: Additional file 1 — Document includes two figures: (1) a screenshot of the PARE web site; and (2) a screenshot of the interactive analysis results page. [file 1471-2105-8-309-S1.doc]

**Supplementary Information for**

***PARE: Comparing Protein Abundance and mRNA Expression Data***


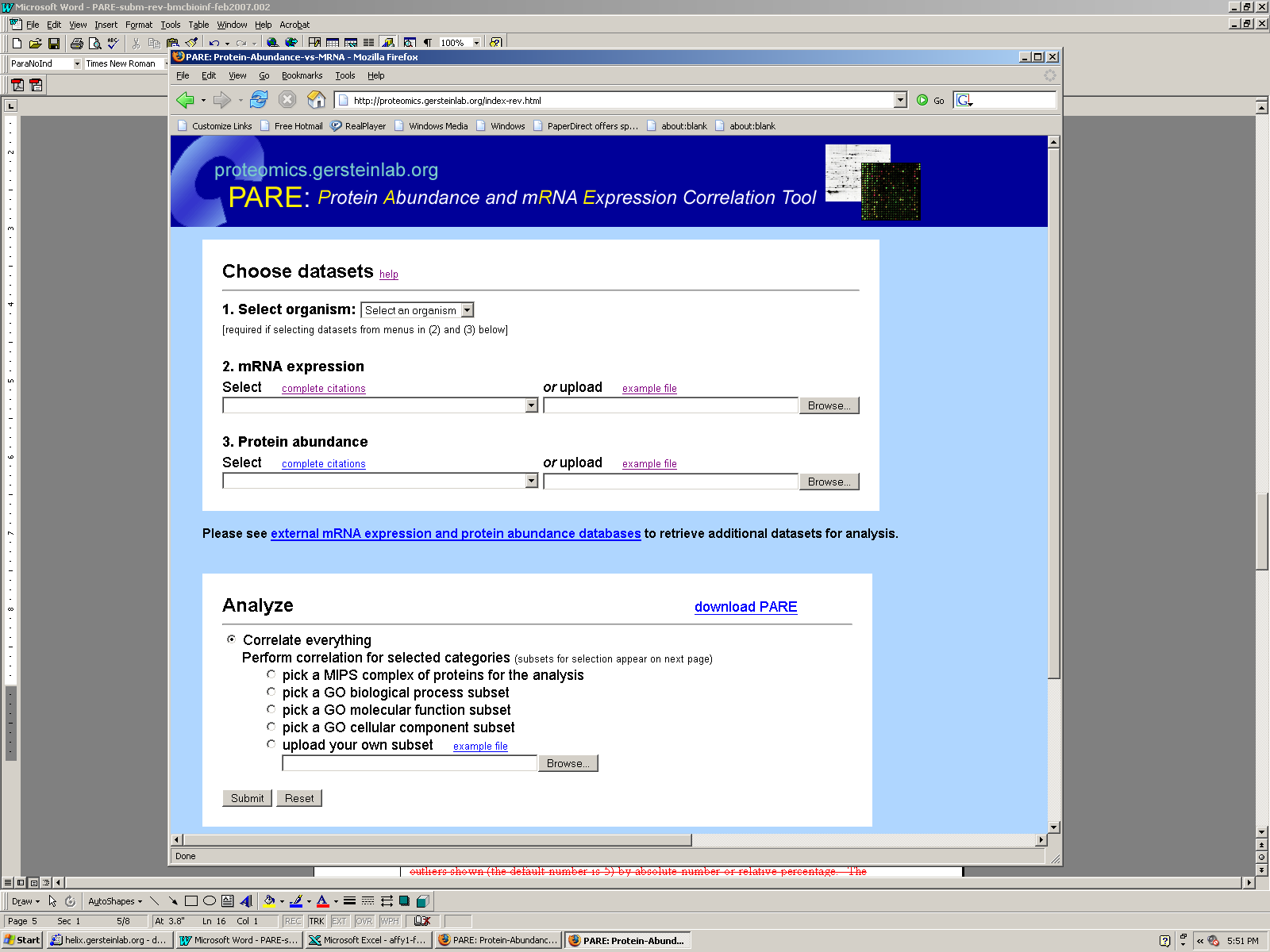


**Fig. 1.**PARE web site


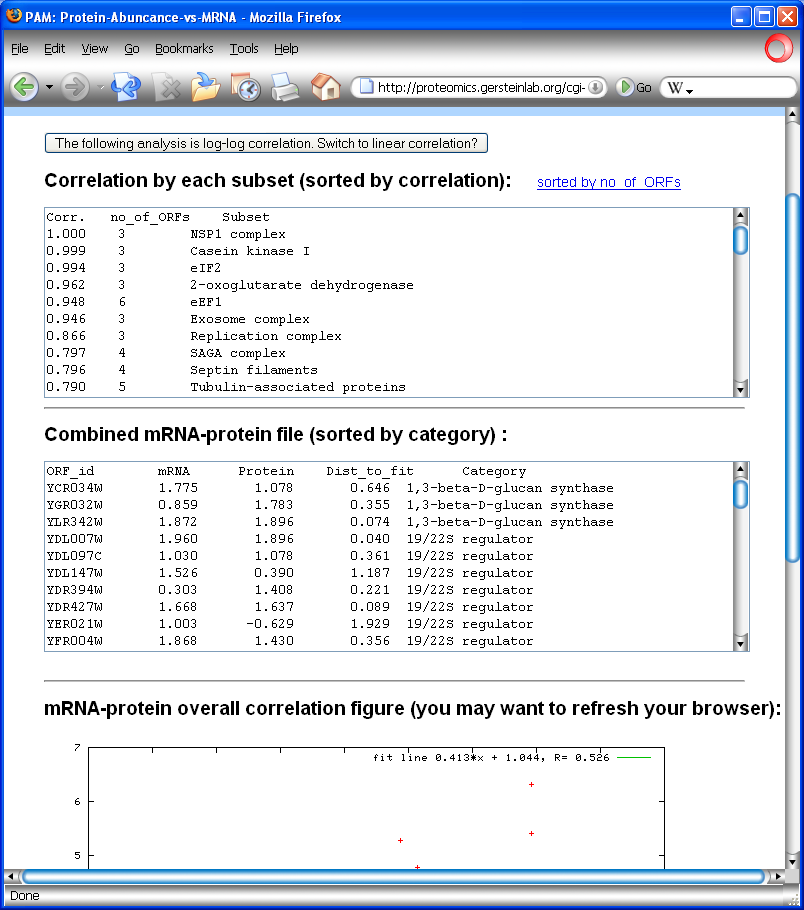

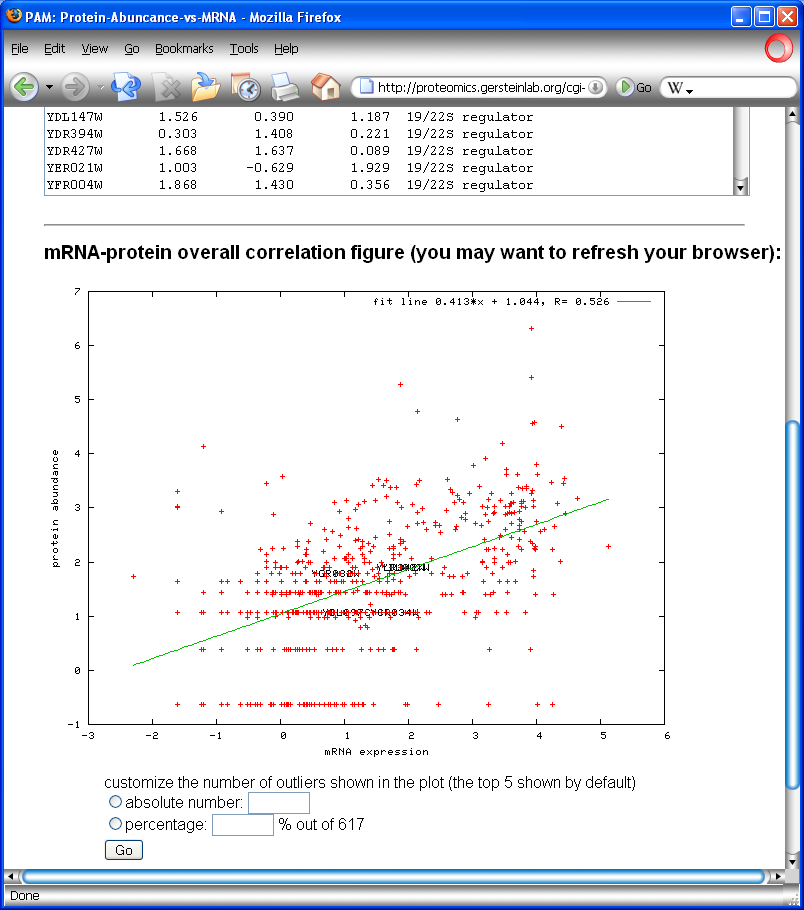


**Fig. 2.** PARE’s interactive analysis result page (MIPS complexes).

**Table 1.** Correlations of the top 5 positively correlated, the top 5 uncorrelated, and the top 5 negatively correlated GO biological process categories (with at least 5 ORFs).

| Correlation | # of ORFs | GO Subset |  |
| --- | --- | --- | --- |
| 1 | 7 | pyruvate metabolism | Highly Correlated Groups |
| 0.959 | 5 | protein complex assembly |
| 0.951 | 8 | protein amino acid glycosylation |
| 0.93 | 22 | response to oxidative stress |
| 0.926 | 5 | Response to drug |
| …… (67 entries) | | | |
| 0.006 | 12 | vesicle-mediated transport | Un-Correlated Groups |
| 0.006 | 39 | ER to Golgi transport |
| -0.021 | 7 | cytokinesis |
| -0.026 | 8 | ribosomal large subunit biogenesis |
| -0.039 | 7 | regulation of transcription, DNA-dependent |
| …… (11 entries) | | | |
| -0.357 | 8 | protein deubiquitination | Highly Anti-correlated Groups |
| -0.406 | 7 | O-linked glycosylation |
| -0.445 | 14 | transcription initiation from Pol II promoter |
| -0.609 | 9 | G1/S transition of mitotic cell cycle |
| -0.691 | 5 | negative regulation of transcription from Pol II promoter |
